# Supplementary material for: Addressing Manufacturability and Processability in Polymer Gel Electrolytes for Li/Na Batteries
Source: Polymers (Basel). 2021 Jun 24;13(13):2093. doi: 10.3390/polym13132093 (PMC8271759; doi:10.3390/polym13132093)
Supplement: Supplementary file 1 [file polymers-13-02093-s001.zip › polymers-1245201-sup-final.pdf]

SUPPLEMENTARY INFORMATION

Addressing manufacturability and processability in polymer gel electrolytes for Li/Na batteries. From gels to highly plasticized polymer electrolytes

Víctor Gregorio, Nuria García, Pilar Tiemblo

Instituto de Ciencia y Tecnología de Polímeros, ICTP-CSIC, Juan de la Cierva 3, 28006 Madrid, Spain.

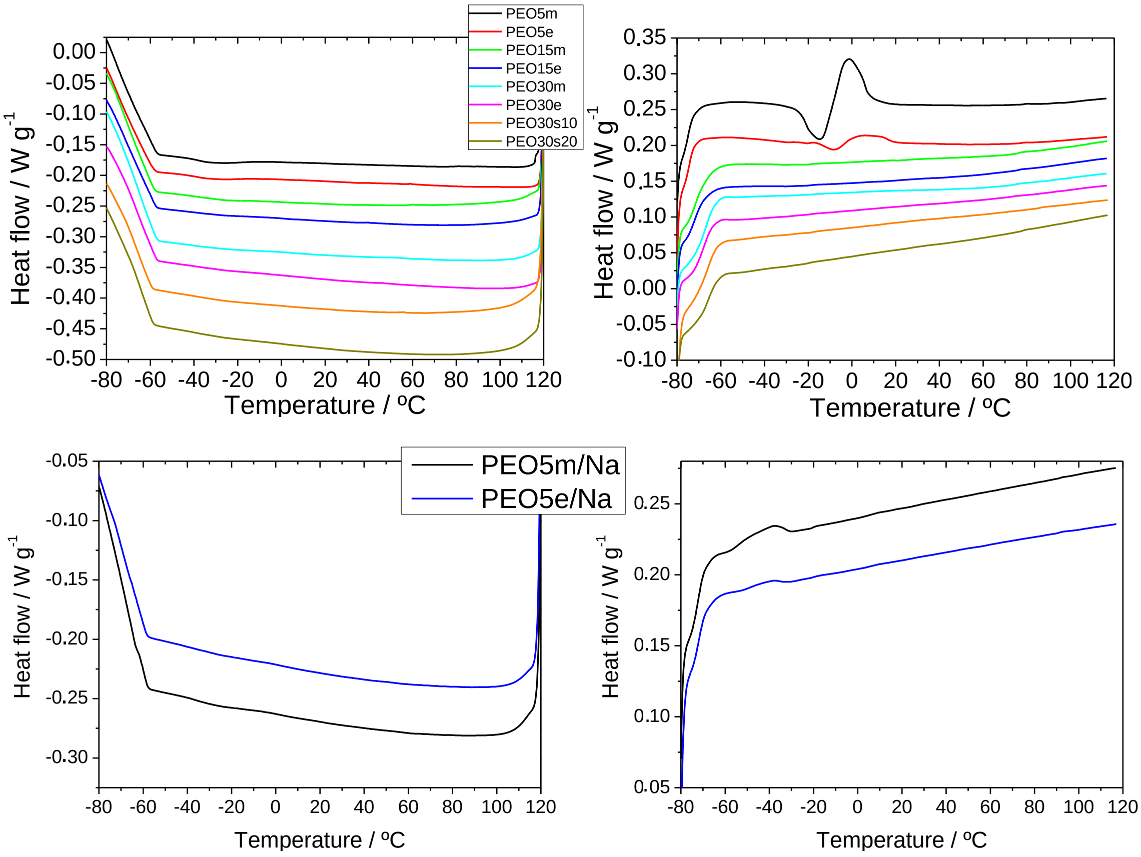

Figure S1: DSC curves of all gel electrolytes prepared on cooling from 120ºC to -80ºC (left) and heating from -80ºC to 120ºC (right).

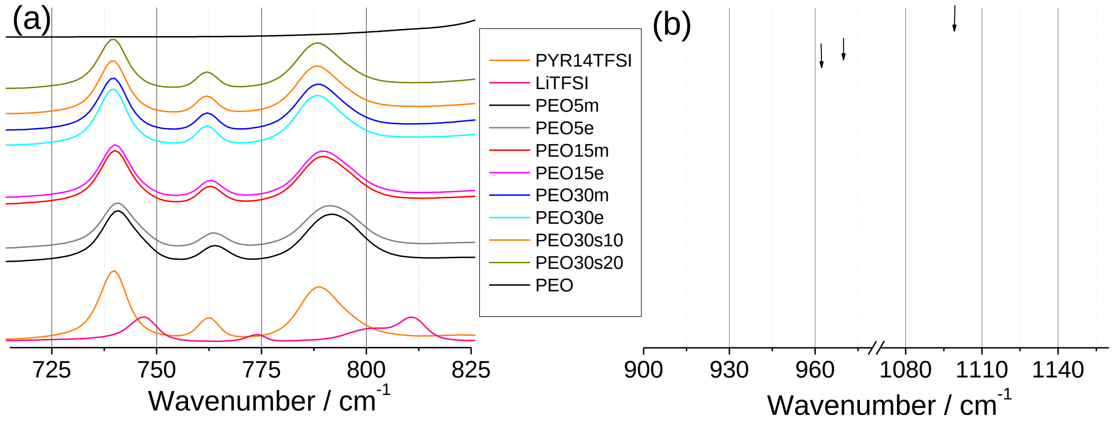

Figure S2: FT-IR of lithium gel electrolytes (a) in the region of the TFSI υ(SN) showing effect of PEO content and preparation method on the relative abundance of free ions, ions pairs and aggregates, and (b) in the region of the PEO methylene deformation (900-970 cm^{-1}) and backbone stretching (1050-1150 cm^{-1})deformations.
