# Supplementary figures and images for: Addressing Manufacturability and Processability in Polymer Gel Electrolytes for Li/Na Batteries
Source: Polymers (Basel). 2021 Jun 24;13(13):2093. doi: 10.3390/polym13132093 (PMC8271759; doi:10.3390/polym13132093)

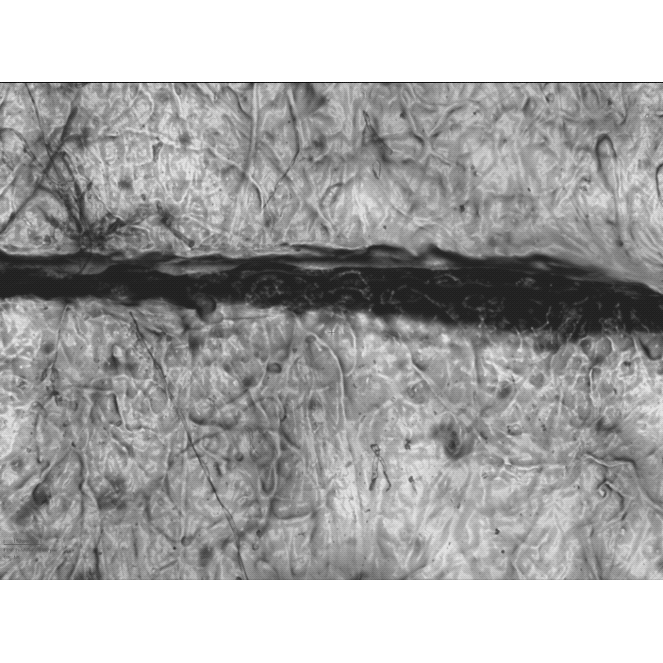

Supplement: Supplementary file 1 [file polymers-13-02093-s001.zip › GIF S1- a series of images taken on the surface of the gel electrolyte PEO30e illustrating its self-healing ability. Images are recorded every 2 minutes and comprise a time span of 40 minutes.gif]
